# Supplementary material for: Stochastic modeling reveals kinetic heterogeneity in post-replication DNA methylation
Source: PLoS Comput Biol. 2020 Apr 10;16(4):e1007195. doi: 10.1371/journal.pcbi.1007195 (PMC7176288; doi:10.1371/journal.pcbi.1007195)
Supplement: S1 File — This file contains supporting text, 26 supplemental figures, and two supplemental tables. (PDF) [file pcbi.1007195.s001.pdf]

Supplemental Information

Stochastic Modeling Reveals Kinetic Heterogeneity in Post-replication DNA Methylation

Luis Busto-Moner[1,2], Julien Morival[3], Honglei Ren[5], Arjang Fahim[3], Zachary Reitz[3], Timothy L. Downing[3,4,5], Elizabeth L. Read[2,4,5,*]

1 Institut Quimic de Sarrià, Universitat Ramon Llull, Barcelona, Spain
2 Dept. of Chemical & Biomolecular Engineering, University of California, Irvine, California, USA
3 Dept. of Biomedical Engineering, University of California, Irvine, California, USA
4 Center for Complex Biological Systems, University of California, Irvine, California, USA
5 NSF-Simons Center for Multiscale Cell Fate Research, University of California, Irvine, California, USA

Supplemental Methods

Monotonic vs. Reversible Methylation Models

We analyzed CpG sites using both the monotonic, 2-parameter model (Eq. 2 in the main text) and the reversible, 3-parameter model (Eq. 5 in the main text). We compared the output of these two models and performed model selection using the Bayesian Information Criterion (BIC), using the formula

$$\mathrm{BIC}_{i,m} = -2l(\hat{\theta}_{i,m}) + p_m \ln(n_i), \tag{1}$$

where i is an individual CpG site index, $l(\hat{\theta}_{i,m})$ is the log-likelihood which is maximized for model m by parameters $\hat{\theta}$ at that given site, p_m is the number of parameters for the model m, (i.e., $m = 2$ or $m = 3$), and n_i is the number of datapoints for site i. We use a threshold ΔBIC of 2 to identify a site as "reversible". That is, we select the reversible, 3-parameter model for site i when:

$$\mathrm{BIC}_{i,2} - \mathrm{BIC}_{i,3} > 2. \tag{2}$$

For Chromosome 1, 14220 out of 876410 sites were identified as reversible with this procedure, or 1.6%. Examples of sites selected for either the monotonic or reversible models are shown in Fig. A.

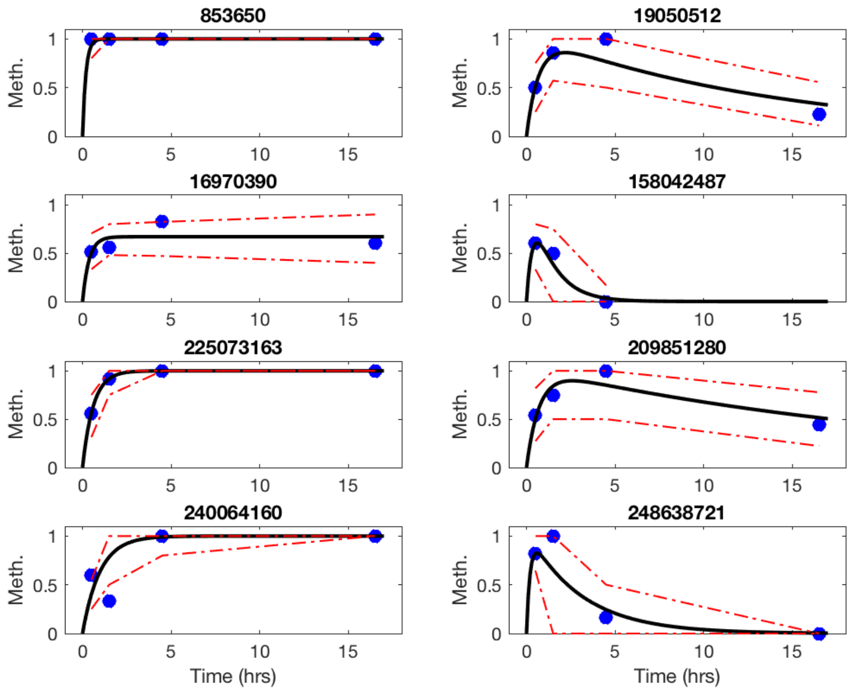

Fig A. Model fits for non-reversible (left) and reversible (right) sites. (Left column) Four sites from Chromosome 1 identified as non-reversible (monotonic), and fit by the 2-parameter model using MLE parameters k, f. Black line: model fit. Blue dots: raw fraction methylation data, averaged per timepoint. Red-dashed lines: 95% confidence intervals from the fitted model, accounting for the experimental number of reads obtained for the site at each timepoint. (Right column) Similar; four sites from Chromosome 1 identified as reversible, and fit by the 3-parameter model using MLE parameters k_1, k_2, f. Each panel is labeled with the SiteID from Chr1.

Choice of Sites to Retain in Analysis

In the experimental Repli-BS dataset, read-depth measured at each CpG site and each timepoint is highly variable. In general, higher read-depth (more samples) leads to higher confidence in estimated parameters. We compared two methods of filtering sites: using a read-depth-based cutoff, and using a confidence-interval-based cutoff.

In the first method (results presented in Main Text and all supplementary figures, unless otherwise noted), all CpG sites are subjected to a cumulative read-depth cutoff of $N = 15$, where at least 10 reads must be acquired at time 0, and at least 5 over subsequent three timepoints. This leads to retaining approximately 40% of CpGs genome wide. A drawback of this method is that statistically it leads to different stringency in different kinetic regimes, since the information gain from reads at different timepoints depends on the kinetics at that site.

Alternatively, we developed a confidence-interval-based cutoff to retain sites only when the width of the Confidence Interval (CI), as estimated by the Profile Likelihood method, is narrower than some threshold. For this analysis, we chose to use CIs on k. For many CpGs, it is not possible to estimate the full width of the 95% CI, since the fast kinetics are not constrained by the experimental timepoints, as described in the Main Text. This poses a challenge to determining a uniform CI-cutoff that can be used across all CpGs. Thus, we estimate the CI half-width (CI_{HW}). For fast sites where the

upper CI limit is not identifiable, we use $CI_{HW} = \log\hat{k} - \log CI_{95}^{-}$, or the difference between the ML estimated k value and the lower 95 CI limit. For sites where the full 95 CI is identifiable, we use $CI_{HW} = (\log CI_{95}^{+} - \log CI_{95}^{-})/2$, or the average width of the upper and lower sides of 95 CI interval.

Choosing variable CI_{HW} thresholds, we find that the number of CpGs retained varies, but that qualitative fitted parameter distributions and correlation functions remain largely unchanged. Results are shown in Fig. B.

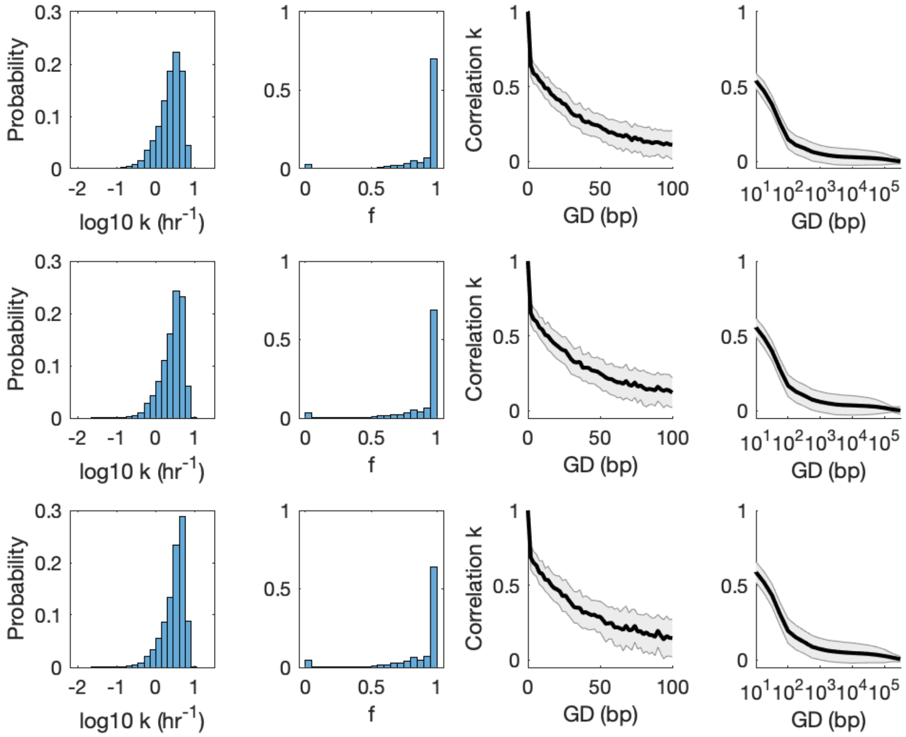

Fig B. Different Choice of Confidence-Interval-based threshold for retaining CpGs in analysis, Chr 1 (Top row) $CI_{HW} = 0.45$, $N_{CpGs} = 1465700$, median cumulative Read-Depth=21. (Middle row) $CI_{HW} = 0.35$, $N_{CpGs} = 999113$, median Read-Depth=23. (Bottom row) $CI_{HW} = 0.3$, $N_{CpGs} = 695616$, median Read-Depth=24.

MLE Validation

Statistical inference of f and k for individual CpG sites required an assessment of the accuracy of such estimation. To do so, $1.5 \cdot 10^4$ sites were each assigned known values of fraction methylation (f_{a_i}) and remethylation rate (k_{a_i}), and remethylation kinetics were simulated *in silico* according to our two-parameter model. Then, MLE was used to infer for each site a fraction methylation (f_{in_i}) and remethylation rate (k_{in_i}), which could be compared to f_{a_i} and k_{a_i}, respectively, so to determine the accuracy of the inference.

To perform the simulation, each site i was assessed the probability of being remethylated at each time-point, j = [0.5, 1.5, 4.5 and 16.5 hr], according to f_{a_i} and k_{a_i} (p_{ij}, see Equation 2 in the Main Text). Also, the number of reads each site would display at each timepoint (n_{ij}) was sampled from the experimental values of Chr1. Then, for a

given site i at the time point j, n_{ij} random numbers from 0 to 1 were generated, and compared to p_{ij}. All random numbers below p_{ij} were considered as methylated, while the rest unmethylated. This was repeated for the other 3 time-points, and all sites, resulting in $1.5 \cdot 10^4$ sites, with methylated and unmethylated reads at the 4 time-points according to their assigned kinetic parameters. Hence, this *in silico* data could be analyzed using MLE, and infer for each site f_{in_i} and k_{in_i}, which could subsequently be compared to the "ground-truth" values, that is the assigned, f_{a_i} and k_{a_i}.

In general, we observe how MLE is able to qualitatively recover assigned k_{a_i} values (Fig. C, A Top). However, when a significant fraction of sites are assigned k_a values beyond the established lower bound (approx. 10 hr^{-1}) k_{in}-distributions appear to be abruptly trimmed (Fig. C, A Middle), for limited time resolution do not allow us to infer rates faster than this limit. Finally, when using more complex distributions, such as the combination of two lognormal functions, MLE allows the recovery of those shapes with relative accuracy (Fig. C, A Bottom).

Also, using a uniformly populated discrete distribution comprising 15 values from 0.03 to 100 hr^{-1} to assign k_a, it is observed that the accuracy of the MLE inference of the remethylation rate constant depend on the magnitude of the assigned k. k-values lower than 0.32 hr^{-1} are inferred with increasing uncertainty (Fig. C B Top), and values faster than 10 hr^{-1} are again assigned to our upper limit. However, our method can accurately estimate a wide range of values, from 0.5 to 5 hr^{-1}. In the case of f, a discrete distribution comprising 10 values from 0 to 1 was used to assign f_a values. It is observed that values of 0 or 1 are assigned with greater accuracy than intermediate values, especially in the $(0, 0.5)$ interval (Fig. C B Bottom).

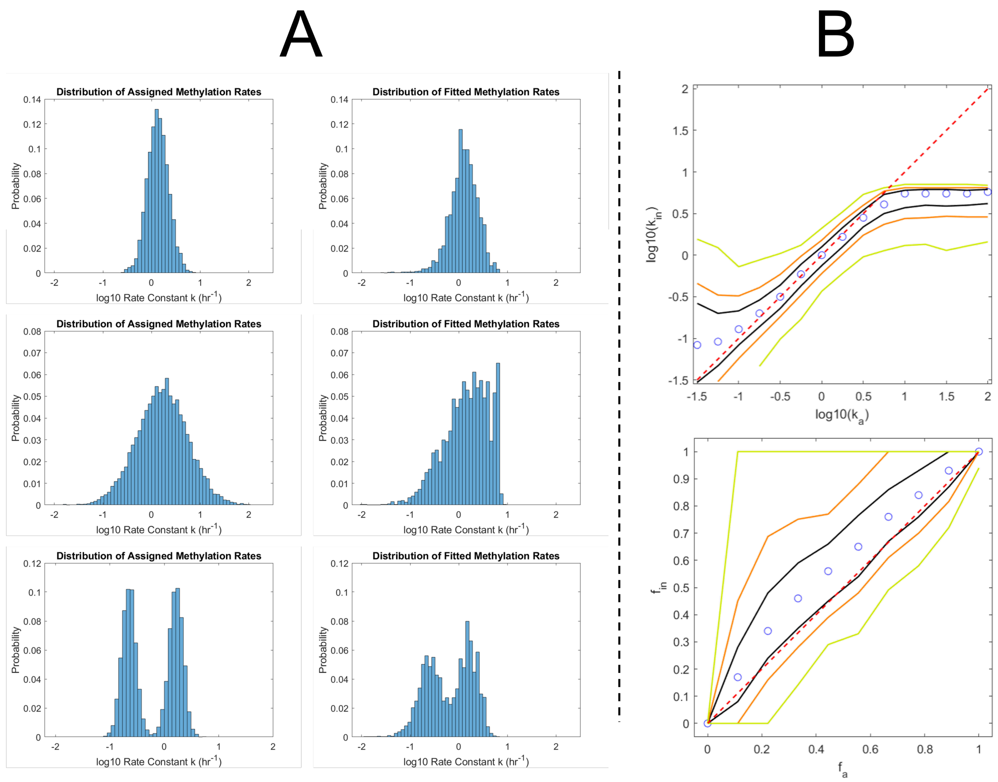

Fig C. Validation of MLE k and f estimation: A: Different lognormal distributions of k_a (left) were used to test MLE ability to recover the same distributions when inferring k_{in} (right). Top: lognormal distribution with mean=0.3 and standard deviation=0.5. Middle: lognormal distribution with mean=0.5 and standard deviation=1.3. Values of k higher than MLE upper limit of 10 hr^{-1} cannot be inferred. Bottom: Sum of 2 lognormal distributions with mean= -1.5 and 0.4 respectively, and standard deviation= 0.3. Assigned f_{a_i} values were sampled from Chr1 inferred f values. **B:** Distributions of inferred parameters k_{in} (Top) and f_{in} (Bottom) when assigning discrete distributions of f_a and k_a. Assigned values lie onto the red-stripped line, while for the distributions of inferred parameters, the median (blue circles), the 50th percentile (black lines), the 75th percentile (orange lines), and 95th percentile (green lines) are represented. For f-analysis, assigned values of f_a ranged from 0 to 1, while k_a values were sampled from fitted k values in Chr1. For k-analysis, assigned k_a values spanned from 0.03 to 100 hr^{-1}, and assigned f_a were sampled from Chr1 inferred values of f.

Effect of the number of reads on the accuracy of the estimation

MLE validation allowed us to analyze the effect of the number of reads, or read-depth (RD), on the inference of remethylation rates. RD shows great variability along a chromosome, with some sites displaying more than 100 reads in total, while others hardly contained more than 5. On average, however, most sites displayed 5 reads at t=0.5, and 5 more at other time-points. Intuitively, statistical inference of poorly covered sites was expected to be more inaccurate than those with more reads. For this reason, we included into the MLE method a restriction regarding the RD at t=0.5 (RD_{t_0}), and the total RD for the rest of timepoints (RD_{later}). Any site with less reads would be disregarded, for it was considered that it did not contain enough reads to be analyzed. However, the more restrictive the method was, the larger the fraction of sites

that were neglected. Therefore, we wanted to assess to what extent the RD could affect the accuracy of the remethylation rate inference, so as to reach a compromise between the quality of sites in terms of their sampling, and the quantity of sites that were perserved.

To that end, a set of 6000 *in silico* sites were assigned remethylation rates from a discrete and uniform distribution of 6 values from 0.56 to 10 hr^{-1}, and fitted using increasingly restrictive conditions in terms of RD_{t_0} and RD_{later}. For the less restrictive conditions (RD_{t_0}=0 and RD_{later}=0), the average relative error was around 40%, while being less than 32% for the most restrictive method, RD_{t_0}=10 and RD_{later}=10, (Fig D A Left). Accordingly, the fraction of sites in Chr1 which were disregarded by MLE with increasing restrictiveness amounted to 75% for the most restrictive conditions (Fig D A Right). Therefore, MLE is proven to be suitable to estimate the order of magnitude the remethylation rate constant of a given site lies in, rather than estimating the exact value with accuracy.

In that sense, when observing the mean square error (MSE) of f and k values obtained when applying MLE on a subset of sites in Chr1, using increasing number of reads, we observed how more reads contributed to a linear reduction in the MSE in terms of k (Fig D B Top). However, when representing log10(k), which provides information regarding the order of magnitude of k, the MSE reached a plateau around 15 reads (combining RD_{t_0} and RD_{later}), and that more reads did not contribute to a significant improvement (Fig D B Bottom). After this analysis, RD_{t_0} of 10 and a RD_{later} of 5 were chosen as suitable conditions that allowed that sites with remethylation rates ranging from 0.56 to 10 h^{-1} were inferred, on average, with less than a 32% of relative error, and preserving, on average, 40 % of the CpG sites of each chromosome.

Regarding $f-$estimation, f_a values were sampled from WGBS measurements from Chr1 in arrested HUES64 cells [1], and k_a were sampled from fitted k values in Chr1. It was determined that fraction methylation could be on average inferred with a $\pm$0.1 in terms of absolute error.

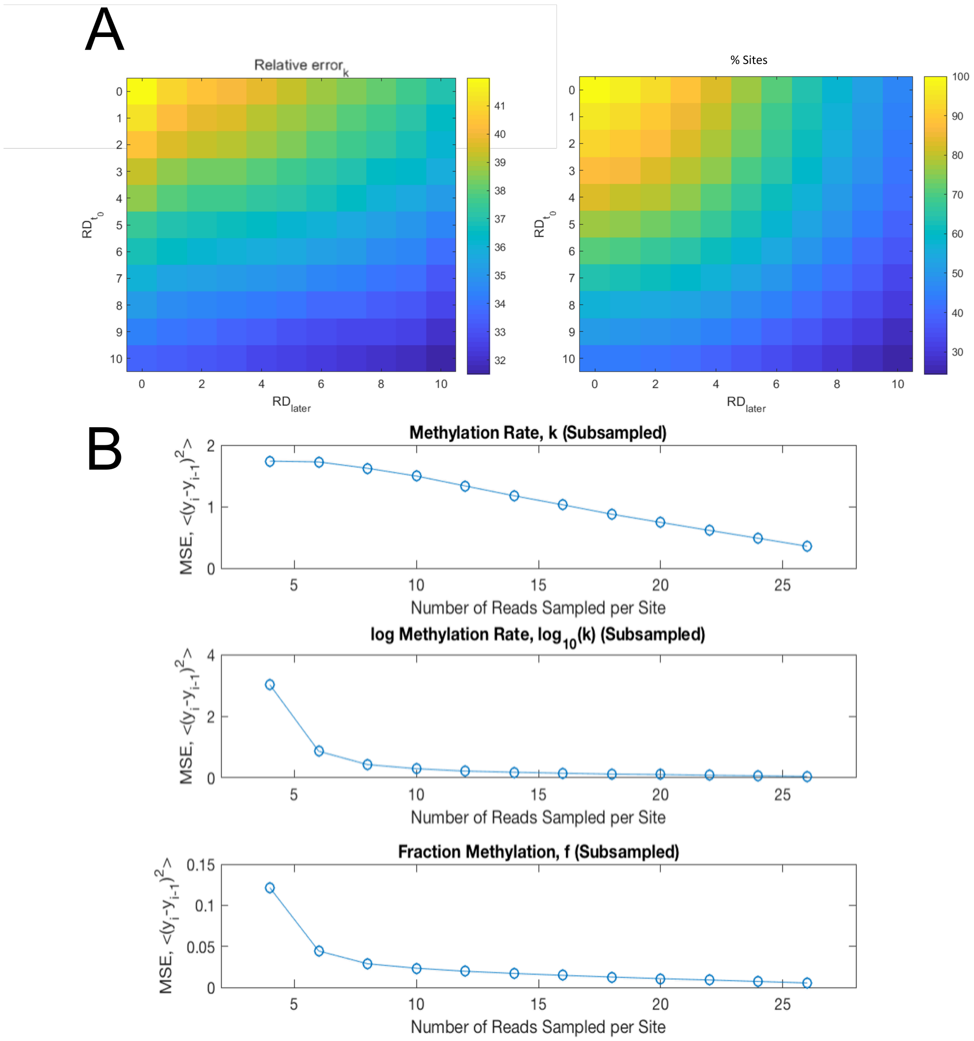

Fig D. Validation of MLE k and f estimation: A: Effect of the Read-depth on the average relative error of k-inference of those sites assigned k_{a_i} values from 0.56 to 10 hr^{-1} (left) and fraction of Chr1 sites that fulfill different read-depth restrictions in terms of RD_{t_0} and RD_{later} (right). **B:** Effect of the Read-depth on the mean-squared error for k (top), $log10(k)$ (middle), and f (bottom). The analysis was performed selecting 121,000 sites from Chr1 that had at least 30 reads (>= 20 at time 0, >= 10 later). From this subset, increasing number of reads were sampled [2,4,...,N-2,N,N+2,...,26) for each site, without replacement, and MLE was performed to fit f and k. The mean squared error of estimates for k, $log10(k)$, and f was determined comparing f and k values of a given subset of fitted sites sampling N reads with f and k inferred after sampling N-2 reads.

Effect of the Bayesian Prior

In order to test the robustness of the two-parameter exponential model in terms of the value of inferred k and f values, they were compared to the results of using a Bayesian Prior method that could incorporate previous information regarding f. The major difference in the estimation method between these results and those of the main text was that, while in the main text no specific assumptions were made on the values of the

parameters, here WGBS experiments in arrested cells were assumed to be informative on f parameters. These independent experimental estimates were included in the calculation of the likelihood function as priors according to Bayes formula. However, note that, for the main text results, although a prior distribution on the parameters was not defined explicitly, priors were implicitly included by construction of the parameter space in calculation of the likelihood surface. That is, f technically has a uniform prior from [0,1], and k has uniform (in log10 space) prior from 10^2 to 10.)

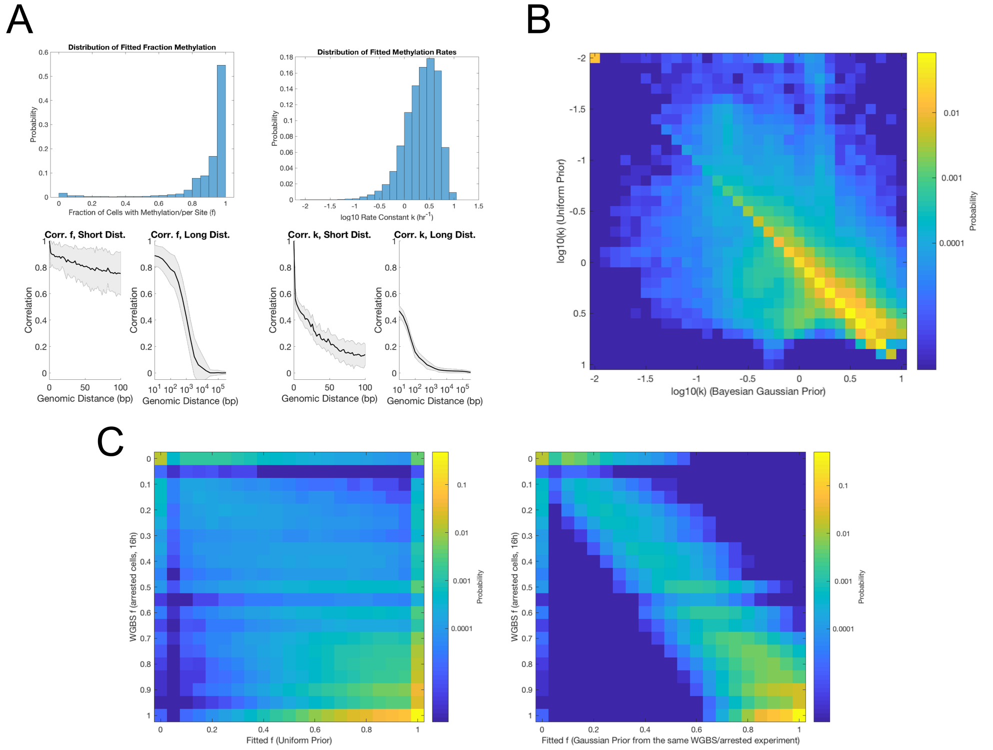

Fig E. Effect of the Bayesian Prior A: Results from Bayesian Parameter Estimation. (Top): Distributions of fitted parameters f (left) and k (right), (Bottom): Correlation of fitted parameters with genomic distance. B: Correlation between k parameter values resulting from two different inference approaches: (y-axis): a uniform prior on f; (x-axis): a strict Gaussian prior on f from WGBS/arrested data. Heat map shows the log-probability of the two distinct approaches to yield values corresponding to a given gridspace. The overall correlation coefficient between the set of individual CpG rate estimates given by the two different approaches was 0.8428. C: Correlation between f at individual CpG sites estimated in two ways: WGBS experiments in arrested cells at 16h, and fitted values from Repli-BS data and our stochastic model/inference approach. Heat maps show the log-probability of the two distinct approaches to yield values corresponding to a given gridspace. (Left): For a uniform prior on f, some correlation between fitted f and WGBS f was apparent. The overall correlation coefficient was 0.6404. (Right): For a prior based on the same dataset, the correlation increases up to 0.9354.

Overall, we find that results were qualitatively consistent between the two inference approaches. Some quantitative differences were seen, but the general results were the same. Specifically, the general shapes and variances of inferred parameter distributions were insensitive to the design of the prior distribution on f. The correlation of k with

genomic distance was qualitatively similar in both cases (though the correlation appears quantitatively reduced overall in the experimental-prior case). Also, the correlation of f with genomic distance was increased by the experimental prior, as expected. While k-estimates on individual sites were affected by the design of the f-prior, there was a high degree of correlation (.84) between individual estimates from both methods.

Effect of Including Experimental Error Estimates

The probability of a methylated read (denoted '1') to be present on the nascent strand at a time t post-replication can be extended to account for the experimental errors. Namely, given a false-positive rate E_p (the probability of a false methylation count) and a false-negative rate E_n (the probability of a false non-methylation count), the probability of observing a methylated read is described in Eq. 6 (Main text).

We compared the results of MLE of the parameters from Repli-BS data using the original formula (no explicit accounting of experimental error) to results using the extended formula with error, above. In the absence of quantified error values for the specific experimental system, we tested a range of error estimates, and found no significant effect on the distributions of inferred parameters chromosome-wide, though some individual site-estimates are impacted (Fig. F).

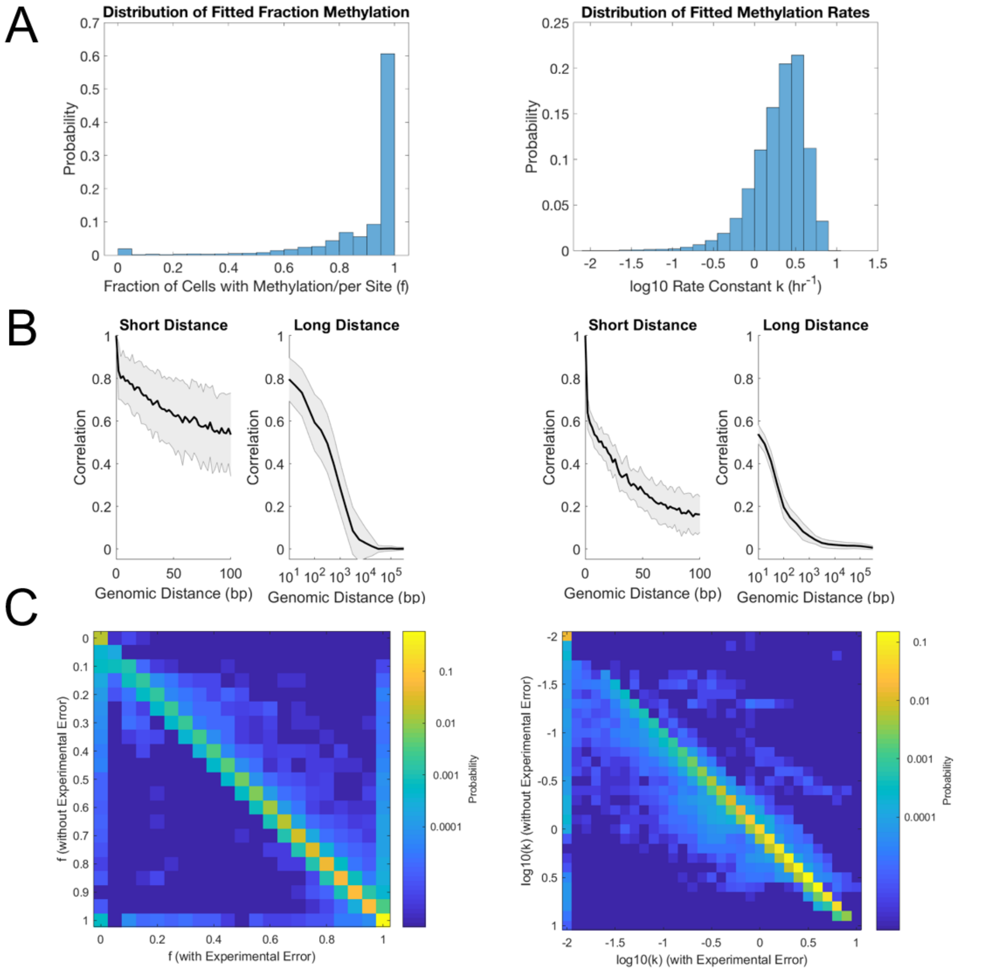

Fig F. Inclusion of experimental error estimates impacts some individual CpG-site estimates, but has minimal impact on MLE parameters chromosome-wide A: Distribution of fitted f (Left) and k (right) values, using MLE with the likelihood function incorporating experimental error (false-positive and false-negative rates). Here, the false-positive rate E_p was assumed to be 1%, and the false-negative rate E_n was assumed to be 0.1%. The largest source of error is assumed to occur in bisulfite conversion efficiency. Since this is estimated to be arround 99% or higher, E_p is estimated at 1%. Smaller sources of error in sequencing, base-calling can affect both E_p and E_n. B: Correlation of fraction methylation f (Right) and remethylation rates k (Left) with GD including the experimental error. C: Correlation of inferred f and k-values with and without experimental error. Overall correlation between the two sets of estimates is 0.9862 for f and 0.9967 for k.

Effect of Including Time as a Random Variable

An alternative method to treat the 0-hour-post-pulse as $t = 0.5$ hours post-replication (and so on for the other experimental timepoints), is to treat post-replication time as a uniformly distributed random variable over the interval of one hour (the duration of the BrdU pulse). Hence, if in our previous model t was defined as:

$$t \in \{0.5,\ 1.5,\ 4.5\ , 16.5\} \tag{3}$$

now each timepoint t_j will be a random variable, uniformly distributed following:

$$p_t(t) = \begin{cases} 0 & t < t_j - \frac{1}{2} \\ 1 & t_j - \frac{1}{2} < t < t_j + \frac{1}{2} \\ 0 & t > t_j + \frac{1}{2} \end{cases} \tag{4}$$

hence assuming that replication initiated anytime within the 1-hr BrdU pulse window. To compute $p(1|k_i, f_i, t)$ for each site i at each timepoint j (See Eq. 2 in the Main Text), we use the expected value $\langle p\,(1|k_i, f_i, t_j)\rangle$, where t is a uniform random variable between $[t_j - \frac{1}{2}, t_j, t_j + \frac{1}{2}]$:

$$\langle p\,(1|k_i, f_i, t)\rangle = \int_{t_j-\frac{1}{2}}^{t_j+\frac{1}{2}} p_t(t) \cdot p\,(1|k_i, f_i, t)\, dt = f_i + \frac{f_i}{k_i}(e^{-k_i(t_j+\frac{1}{2})} - e^{-k_i(t_j-\frac{1}{2})}) \tag{5}$$

We compared the results of MLE of the parameters from Repli-BS data using the original method (no random time-variable) to results using time as a random variable. Overall, we found no significant effect on the distributions of inferred parameters chromosome-wide, though some individual site-estimates are impacted (Fig. G).

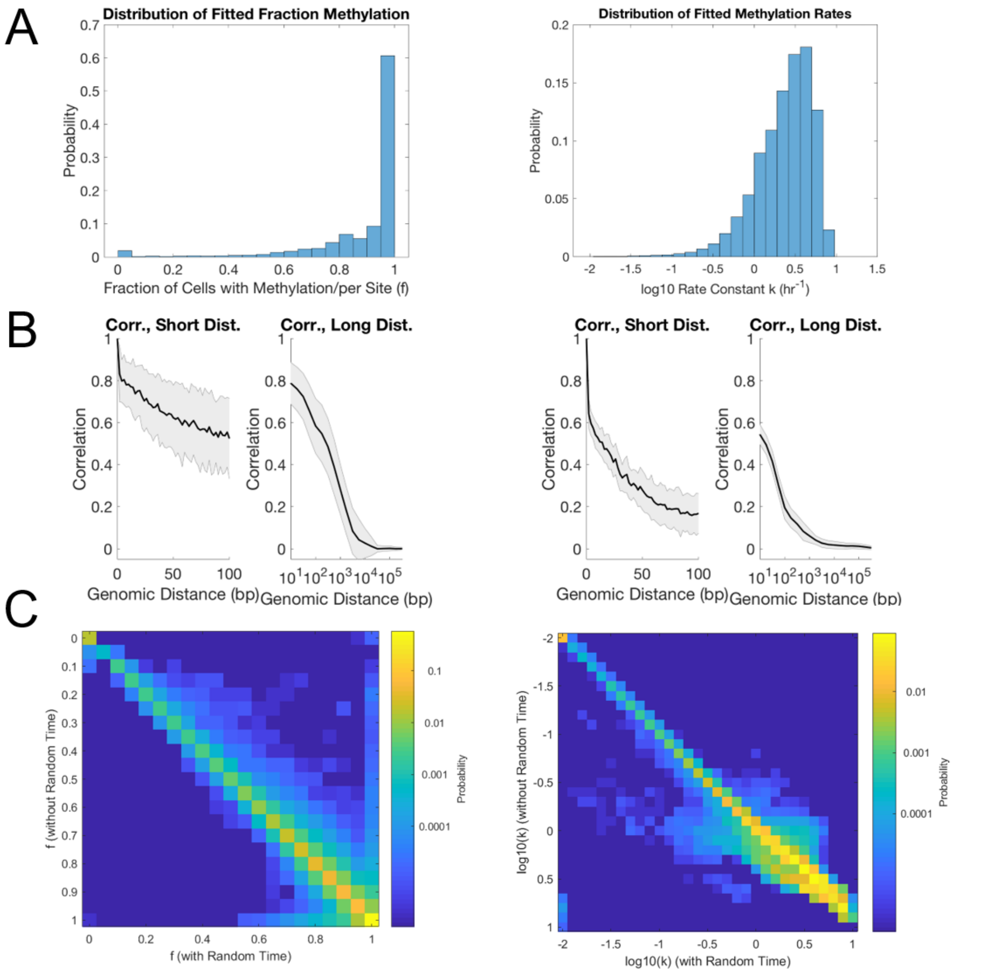

Fig G. Inclusion of time as a random variable impacts some individual CpG-site estimates, but has minimal impact on MLE parameters chromosome-wide. A: Distribution of fitted f (Left) and k (right) values, using a random time-variable. B: Correlation of fraction methylation f (Right) and remethylation rates k (Left) with GD using a random time-variable. C: Correlation of inferred f (Left) and k(Right)-values with and without using time as a random variable. Overall correlation between the two sets of estimates is 0.9930 for f and 0.9540 for k.

Single-basepair-level stochastic enzyme-kinetic models

Distributive mechanism

The distributive model, which serves as a common backbone for the Processive and the Collaborative models, is based on a Compulsory-Order Ternary-Complex Mechanism (COTCM), by which DNMT1 (E) first binds the hemimethylated CpG (h) to form the Eh complex. Then, SAM (S) can form a ternary complex named ESh. Species m stands for the methylated CpG, and Q for a SAM molecule which, after methylation, has lost its methyl group. In order to assess the value of the forward and reverse rate constants for the first two binding reactions 1 and 2, being the first the binding and unbinding of E with h, whose rate constants are presented as k_{1f} and k_{1r} respectively, and the second the incorporation of S to the Eh complex to form ESh and its dissociation, presented as k_{2f} and k_{2r} respectively, four mathematical relationship can

be derived from the classical model COTCM [2]. When doing so, it has been assumed that after ESh formation, methylation and enzyme turnover can take place in one irreversible and limiting step, whose rate constant is k_3 [3] (See Fig. 2 in the Main Text).

Therefore, at t=0, the rate of the reaction can be defined as:

$$v_0 = \frac{d[m]}{dt} = k_3[EhS] \tag{6}$$

For the purpose of this analysis, we assume that that all intermediates are in the steady state, thus being:

$$\frac{d[EhS]}{dt} = k_{2f}[Eh][S] - (k_3 + k_{2r})[EhS] = 0 \tag{7}$$

$$\frac{d[Eh]}{dt} = k_{1f}[E][h] + k_{2r}[EhS] - (k_{1r} + k_{2f}[S])[Eh] = 0 \tag{8}$$

Also, that $[S] \approx [S]_0 >>> [EhS]$, where $[S_0]$ is the initial concentation of SAM. On the other hand, the total concentration of enzyme, $[E]_0$, can be defined as:

$$[E]_0 = [E] + [Eh] + [EhS] \tag{9}$$

From Eq. 7, it can be derived that:

$$[EhS] = \frac{k_{2f}[S]}{k_{2r} + k_3}[Eh] \tag{10}$$

For the sake of simplicity, we will group some terms of Eq. 10 into p:

$$p = \frac{k_{2f}[S]}{k_{2r} + k_3} \tag{11}$$

From Eq. 8, it can be derived that:

$$[Eh] = \frac{k_{1f}[h]}{k_{1r} + (k_{2f} - \frac{k_{2r}k_{2f}}{k_3+k_{2r}})[S]}[E] \tag{12}$$

Again, for the sake of simplicity we will group some terms of Eq. 12 into a term named q:

$$q = \frac{k_{1f}[h]}{k_{1r} + (k_{2f} - \frac{k_{2r}k_{2f}}{k_3+k_{2r}})[S]} = \frac{k_{1f}k_{2f}k_3[h]}{k_{1r}(k_3 + k_{2r}) + k_{2f}k_3[S]} \tag{13}$$

Thus, given Eq. 9:

$$[E]_0 = [E] + q[E] + pq[E] \tag{14}$$

Therefore:

$$[E] = \frac{[E]_0}{1 + q + pq} \tag{15}$$

We can now define the initial velocity of the reaction v_0 as:

$$v_0 = k_3 \cdot p \cdot q \cdot \frac{[E]_0}{1 + q + pq} \tag{16}$$

Rearranging some terms in Eq. 16:

$$v_0 = \frac{k_3[E]_0}{\frac{1}{pq} + \frac{1}{p} + 1} \tag{17}$$

If we now retrieve the definitions of p and q (Eq. 11 and 13, respectively),we obtain:

$$v_0 = \frac{k_3[E]_0}{\frac{k_{2r}+k_3}{k_{2f}[S]} \cdot \frac{k_{1r}(k_3+k_{2r})+k_{2f}k_3[S]}{k_{1f}[h](k_{2r}+k_3)} + \frac{k_{2r}+k_3}{k_{2f}[S]} + 1} \tag{18}$$

Simplifying Eq 18:

$$v_0 = \frac{k_3[E]_0}{\frac{k_{1r}}{k_{1f}} \cdot \frac{k_{2r}+k_3}{k_{2f}} \cdot \frac{1}{[S][h]} + \frac{k_3}{k_{1f}}\frac{1}{[h]} + \frac{k_{2r}+k_3}{k_{2f}}\frac{1}{[S]} + 1} \tag{19}$$

From Eq 19, we can define 4 mathematical expressions as:

$$K_{mh} = \frac{k_3}{k_{1f}} \tag{20}$$

$$K_{mS} = \frac{k_{2r} + k_3}{k_{2f}} \tag{21}$$

$$K_{ih} = \frac{k_{1r}}{k_{1f}} \tag{22}$$

$$k_{cat} = k_3 \tag{23}$$

leaving Eq 19 as:

$$v_0 = \frac{k_3[E]_0}{\frac{K_{ih}K_{mS}}{[S][h]} + \frac{K_{mh}}{[h]} + \frac{K_{mS}}{[S]} + 1} \tag{24}$$

The value of these 4 parameters (K_{mh}, K_{mS}, K_{ih}, and k_{cat}) have been extracted from [4]. They reported the Michaelis constants K_{mh} and K_{mS} for the case of recombinant human DNMT1 for both hemimethylated small nuclear riboprotein-associated peptide N (SNRPN) exon-1 and SAM, as well as DNMT1 catalytic turnover k_{cat} and the constant K_{ih}. In the case of K_{mh} and K_{mS}, authors presented values for two different hemimethylated SNRPN exon-1 substrate, one methylated on the upper strand and another on the lower. Since our experiments were based on the remethylation of the whole genome after replication, in which two hemimethylated molecules are present, one with the upper and one with the lower methylated strand, an average value was taken for both. The same strategy was used to determine the value of k_3, that can be directly associated with k_{cat} by Eq 23. The assumed value of K_{ih}, on the other hand, was extracted from the double reciprocal plot obtained by Pradhan et al. when representing different rates of reaction as a function of the concentration of SNRPN exon-1, while keeping SAM initial concentration constant.

Since the value of 5 kinetic constants (k_{1f}, k_{1r}, k_{2f}, k_{2r} and k_3) had to be determined out of 4 mathematical relationships (equations 20 to 23), based on 4 experimental parameters (K_{mh}, K_{mS}, K_{ih} and k_{cat}), the value of either k_{2f} or k_{2r} had to be arbitrarily assessed. Eventually, the value of k_{2r} was determined to be 100 hr^{-1}. Interestingly enough, since the value of the forward process is proportional to the reverse (Eq 21), the effect of varying this arbitrary parameter on the model did not have any significant effect in terms of the kinetics of the model (Data not shown) or k-correlation with genomic distance in the case of the Processive and the Distributive mechanisms (Fig. X and Y).

Parameter	Symbol	Units	Value	Source	Mechanism
Michaelis constant for the binding of Enzyme and SAM	K_{mS}	μM	5.5	[4]	All
Michaelis constant for the binding of Enzyme and h-CpG	K_{mh}	μM	1.3	[4]	All
Michaelis constant derived from COTCM	K_{ih}	μM	2.85	[4]	All
Enzyme turnover	k_{cat}	hr^{-1}	40	[4]	All
Enzyme binding to h-CpG	k_{1f}	$(copy \cdot hr)^{-1}$	0.14*	Eq 20	All
Enzyme unbinding from h-CpG	k_{1r}	hr^{-1}	88.7	Eq 22	All
SAM binding to Enzyme	k_{2f}	$(copy \cdot hr)^{-1}$	0.12*	Eq 21	All
SAM unbinding from Enzyme	k_{2r}	hr^{-1}	100	Arbitrary	All
Methylation reaction	k_3	hr^{-1}	40	Eq 23	All
Nuclear volume	V	pL	1	[5]	All
h-CpG copies in mammal cells	h_0	$copy$	$2.8 \cdot 10^7$	[6]	All
DNMT1 copies in mammal cells	E_0	$copy$	$2.8 \cdot 10^5$	Arbitrary	All
SAM copies in mammal cells	SAM_0	$copy$	$5.6 \cdot 10^7$	Arbitrary	All
Enzyme drop-off from DNA	k_{off}	hr^{-1}	8	[7]	Processive
Enzyme 1-D diffusion coefficient	D	$bp^2 s^{-1}$	10^6	[8, 9]	Processive
Processive diffusion distance limit	$nDist$	bp	3600	[10]	Processive
Recruitment distance-function parameter 1	a	bp	10^4	Arbitrary	Collaborative
Recruitment distance-function parameter 2	b	bp	0.2	Arbitrary	Collaborative
Collaborative recruitment neighbor index limit	nN_{col}	-	200	Arbitrary	Collaborative

*This value corresponds to simulating $N_{sites} = 10^4$ sites, according to Eq 25

Table A. Table of used parameters for the Distributive, Processive and Collaborative models

Other parameters that had to be assessed were the number of CpG-sites in the whole genome (h_0), the number of DNMT1 copies in a cell (100-fold lower than h_0), or the nuclear volume of a mammalian cell (V). The concentration of SAM was set to be 200 times larger than DNMT1, so it was always present in sufficient amounts. Identical values for all these parameters were also used in both the Processive and the Collaborative model. The value of all parameters is displayed in Table A.

Since reaction rate constants k_{1f} and k_{2f} presented $(\mu M \cdot hr)^{-1}$ units, they had to be converted to copy-number units and scaled to the number of sites we were simulating (N_{sites}) following:

$$k_{if_{copy}} = \frac{k_{if_{molar}} \cdot h_0 \cdot 10^6}{N_{sites} \cdot V \cdot N_A} \tag{25}$$

Where $k_{if_{copy}}$ corresponds to k_{1f} or k_{2f} in $(copy \cdot hr)^{-1}$ units, $k_{if_{molar}}$ corresponds to k_{1f} or k_{2f} in $(\mu M \cdot hr)^{-1}$ units, h_0 corresponds to the number of CpG-sites in a cell nucleus, V corresponds to the volume of a cell nucleus, and N_A corresponds to the Avogadro's constant.

Similarly, the number of DNMT1 and SAM copies at the beginning of a simulation (E_i and SAM_i respectively) was determined scaling nuclear values (E_0 and SAM_0 respectively) according to the number of sites that were simulated (N_{sites}):

$$E_i = \frac{E_0 \cdot N_{sites}}{h_0} \tag{26}$$

$$SAM_i = \frac{SAM_0 \cdot N_{sites}}{h_0} \tag{27}$$

Processive mechanism

In the Processive mechanism, DNMT1 can diffuse linearly along DNA towards neighbor CpG-sites after methylation, traveling either upstream or downstream. In order to incorporate diffusion efficiently into the stochastic simulations, we applied a First Passage Time Kinetic Monte Carlo algorithm based on ref [**?**]. The diffusion model uses a 1D lattice with inter-lattice spacing $r = 1$ basepair. Consider an enzyme bound to a CpG at position x_{start} along DNA where it has just catalyzed methylation, with

potential target sites (neighbor hemimethylated sites) at distance d_U and d_D upstream and downstream, respectively, on DNA. The enzyme moves with diffusion coefficient D and can unbind from any site with rate k_{off}. The value of the diffusion coefficient of DNMT1 along DNA was assumed to be in the order of other 1D sliding coefficients of well-known transcription factors such as LacI and p53, reported in [8, 9] (See Table A). A Master Equation is constructed with state space enumerated by the vector $\mathbf{x} = \{x_{start} - d_U, ..., x_{start}, ...x_{start} + d_D, S\}$, which includes all positions x on DNA between and including the nearest upstream hemimethylated neighbor and the nearest downstream hemimethylated neighbor, as well as the state S representing the solution. The enzyme can reach one of three possible exit states: it can eventually reach by diffusion the nearest h neighbor either Upstream or Downstream, or it can unbind to the solution. For the purposes of calculating the First Passage Time Distribution and relative probability of reaching each of these three states, these exit states are considered to be absorbing (only processes into, but not out of, these states are considered). Thus, the full Master Equation comprises N states where $N = d_U + d_D + 2$, and is given by:

$$\frac{\partial P(x,t)}{\partial t} = \frac{D}{r^2} P(x+1,t) \qquad x = x_{start} - d_U \tag{28}$$

$$\frac{\partial P(x,t)}{\partial t} = \frac{D}{r^2} P(x-1,t) \qquad x = x_{start} + d_D \tag{29}$$

$$\frac{\partial P(x,t)}{\partial t} = \frac{D}{r^2} P(x+1,t) + \frac{D}{r^2} P(x-1,t) - (\frac{2D}{r^2} + k_{off}) P(x,t) \tag{30}$$

$$\frac{\partial P(S,t)}{\partial t} = \sum_x k_{off} P(x,t) \tag{31}$$

$$x_{start} - d_U < x < x_{start} + d_D$$

The Master Equation is expressed as an $N \times N$ matrix. The Eignevalues and Eigenvectors of the matrix can be utilized to numerically compute the First Passage Time Densities, given that the enzyme starts at position $_{start}$, according to Gillespie's Eigenvalue Approach. Furthermore, the exit probabilities (the relative probability of exiting to each of the three exit states at time τ, given that the enzyme has not yet left the region before τ, and given that it started at x_{start}) was obtained by numerical integration of the Master Equation to obtain the relative relative flux of probability into the absorbing states at waiting time τ.

Collaborative mechanism

Collaborative recruitment reactions were assigned propensities k_{RecU} and k_{RecD}, which result from weighting k_{1f} with a distance-dependant function (See Eq 16 in the main text). Note that in this case the two sites involved in the recruitment do not have to be contiguous, and no distance restrictions were imposed. Hence, the second DNMT1 copy could virtually be recruited onto any neighboring site. However, this would imply including a large number of recruitment reactions to the model, significantly increasing the computation time. Extensive analysis of different simulations showed that recruitments onto sites further than the 200th neighbor were practically nonexistent, albeit having non-zero propensities. Reactions corresponding to these further recruitments constituted less than 10% of all recruiting reactions, even when performing simulations with the highest CpG_d substrates. In those substrates, CpG sites are all separated by a distance of 2 *bp* (the minimum distance these dinucleotides can be apart), so the propensities of furthest recruitments are maximized. Therefore, with the aim of reducing the computation times, the finite number of recruitments upstream or downstream was set to be $nN_{col} = 200$.

Stochastic simulations

Both the Distributive, the Processive, and the Collaborative mechanisms were used to stochastically simulate DNA maintenance methylation by DNMT1 kinetics in the context of replication, using the Stochastic Simulation Algorithm [11]. Simulated DNA substrates contained N_{sites} CpG sites that could be either hemymethylated (h), and thus undergo remethylation, or unmethylated (u), according to the methylation landscape of arrested HUES64 cells.

In the case of the Distributive mechanism (See Fig 2A in the main text), each site that was h at t=0 could present 4 different states along the simulation: h, Eh, EhS and m. For each time-step, 5 possible reactions could occur (1_f, 1_r, 2_f, 2_r, and 3) on one of the N_{sites} sites. Then, the current state of the system changed according to the stoichiometry of the chosen reaction. The propensities of every reaction for every site were subsequently recalculated, and the process was repeated. To choose the reaction, the site it would occur on and the duration of every interval, two random numbers where generated in every step, following the Gillespie algorithm.

Immediately after time exceeded one of the experimental time-points (0.5, 1.5, 4.5 and 16.5 hr), the whole state of the system was recorded, saving as 'methylated' any site in m, and as 'unmethylated' any site in u, h, Eh or EhS. Eventually, the simulation stopped right after time would exceed 16.5 hr.

Regarding the Processive mechanism, the same procedure was followed, but the number and type of reactions, as well as the possible states a CpG site could adopt were different. A total of 8 reactions could take place, including reactions 1_f, 1_r, 2_f, 2_r, 3, and 6, in addition to the two diffusion reactions, upstream or downstream (See Fig 2B in the main text). The propensities of the two processive reactions (k_{DifU} and k_{DifD} were calculated for every site, according to the distance between it and its two contiguous neighbors. The propensities of hops towards neighbors placed at a distance larger than $3600bp$ where directly set to 0. Moreover, the propensity of impossible hops, such as the one from the first CpG site on the 5' top of the DNA strand towards an hypothetical neighbor upstream, were also assigned a value of zero. The Processive mechanism contemplated 6 possible states for any CpG site, u, h, Eh, EhS, Em and m, being the first four included in the category of unmethylated, while the last two were considered as a methylated read when recording the state of the system at times 0.5, 1.5, 4.5 and 16.5 hr. Aside from these differences, the procedure in terms of simulation was identical to the Distributive mechanism.

For the Collaborative mechanism, a total of $5+2nN_{col}$ reactions could take place, where 5 includes reactions 1_f, 1_r, 2_f, 2_r, and 3 (See Fig. 2 C in Main Text), and nN_{col} stands for the range of neighbors, upstream or downstream the recruiting site, onto which other DNMT1 copies can be incorporated in a collaborative fashion. This way, $nN_{col} = 4$ would mean that a second DNMT1 could be recruited onto the first, second, third or fourth neighbor upstream the site where the first enzyme copy was bound, or onto the first, second, third, or fourth neighbor downstream, generating 8 additional reactions for each site. The propensity of each recruitment reaction onto each neighbor within the nN_{col} range was determined according to the distance between the recruiting and the neighbor hemimethylated CpG sites, and a non-dimensional distance-dependent function (See Eq. 13 in the Main Text). Just like with the Processive mechanism, the propensity of impossible reactions, like DNMT1 recruitment downstream another copy bound at the top 3' of the DNA strand, was set to 0. In the Collaborative model, 5 possible states were contemplated for any site : u, h, Eh, EhS, and m, and the state of the system was again recorded at times 0.5, 1.5, 4.5, and 16.5 hr. While those sites displaying m where considered as methylated reads, any site in u, h, Eh, or EhS was again considered to be unmethylated.

Either using the Distributive, the Processive or the Collaborative mechanism, by

Dataset	N	Pearson	Spearman
Chr 1	876410	-0.0151	-0.1623
Mean, all Chr	10400611	-0.0320	-0.1727
Sim. Trial 1 ground truth	40000	0.011	0.0049
Sim. Trial 1 inferred	40000	-0.0482	-0.0999
Sim. Trial 2 ground truth	40000	-0.0017	-0.0048
Sim. Trial 2 inferred	40000	-0.0451	-0.1030
Sim. Trial 3 ground truth	40000	0.0062	0.0030
Sim. Trial 3 inferred	40000	-0.0492	-0.0939

Table B. Table of computed correlation coefficients (Pearson and Spearman) between ML inferred k and f values. Ground truth simulations show that the MLE procedure introduces correlation (-0.0475 and -0.0989, Pearson and Spearman, respectively, average of 3 trials.) The parameters inferred from data show a more pronounced negative correlation.

repeating the simulation on the same substrate a certain number of times ($NReads$), at the end of the whole process N_{sites} CpG sites were simulated, each with a certain number of methylated reads (m) and unmethylated reads (u) at every time-point. Therefore, each repetition out of $NReads$ can be compared to every experimental read. In that sense, and to replicate experimental conditions, in which sites show a larger number of reads at t=0.5 than the other three time-points, the results of 5 short simulations from 0 to 0.5 hr were added to 5 simulations from 0 to 16.5 hr, thus yielding 10 reads at t=0.5, and 5 reads at 1.5, 4.5, and 16.5 hr.

Eventually, this procedure gave rise to a simulation of the remethylation kinetics of N_{sites} CpG sites. Each site displayed a certain number of methylated and unmethylated reads at times 0.5, 1.5, 4.5 and 16.5 hr, just like the experimental data, but according to any of the 3 possible mechanisms. This way, MLE fitting could be used to infer f and k for each of these simulated sites (f_{model} and k_{model} respectively), and compare their distributions and correlation with GD and CpG_d, to elucidate if mechanistic differences can account for experimental observations.

Supplemental Results

Correlation between inferred k and f values

We find that the inferred k and f values are weakly negatively correlated. However, it is possible for some spurious correlation to be introduced by the MLE fitting procedure, due to the limited read depth. To assess this, we carried out "ground truth" simulations, similar to those described in Fig. C. Ground-truth values of k and f were generated in an uncorrelated manner. Simulated Repli-BS data was then produced with the timepoints and per-site-per-time read-depths chosen by sampling from the true data read-depths for Chr1. k and f were inferred from the simulated data and correlation was assessed (see Table B).

Remethylation rates for Chr 13 to 22

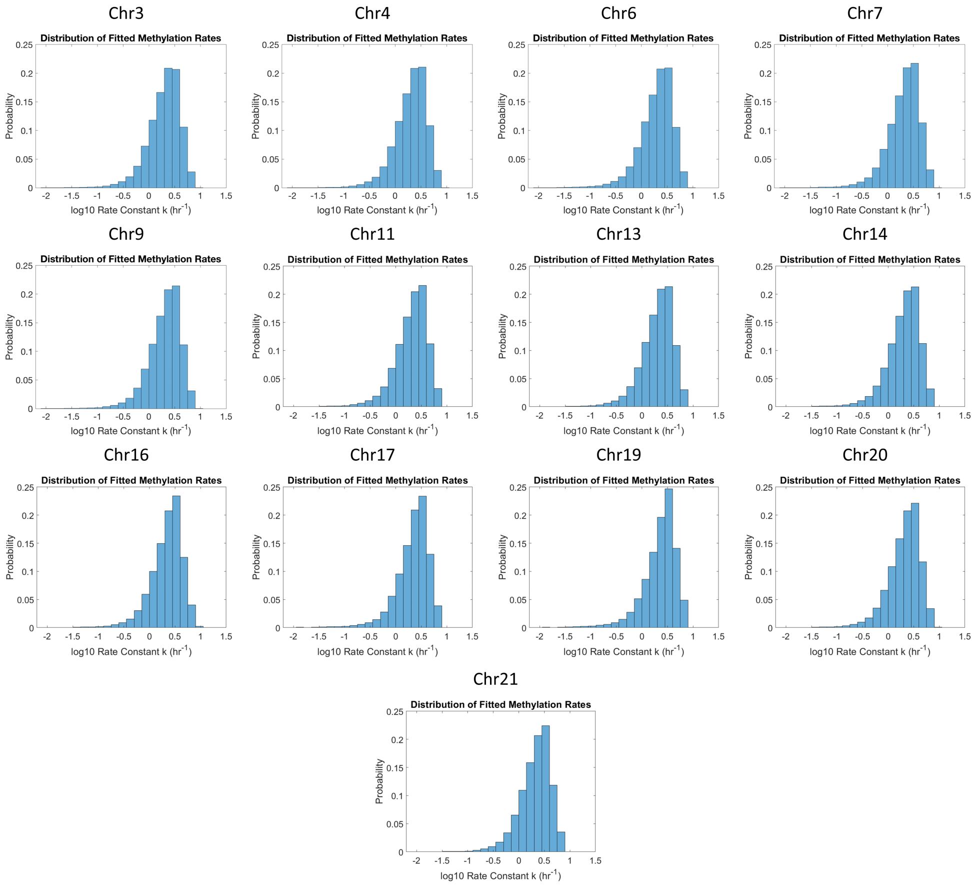

Fig H. Histogram of remethylation rates, k, for Chromosomes 3, 4, 6, 7, 9, 11, 13, 14, 16, 17, 19, 20, and 21. Histograms are normalized by probability. (Results for other chromosomes are shown in Main Text.)

Steady-state fraction methylation

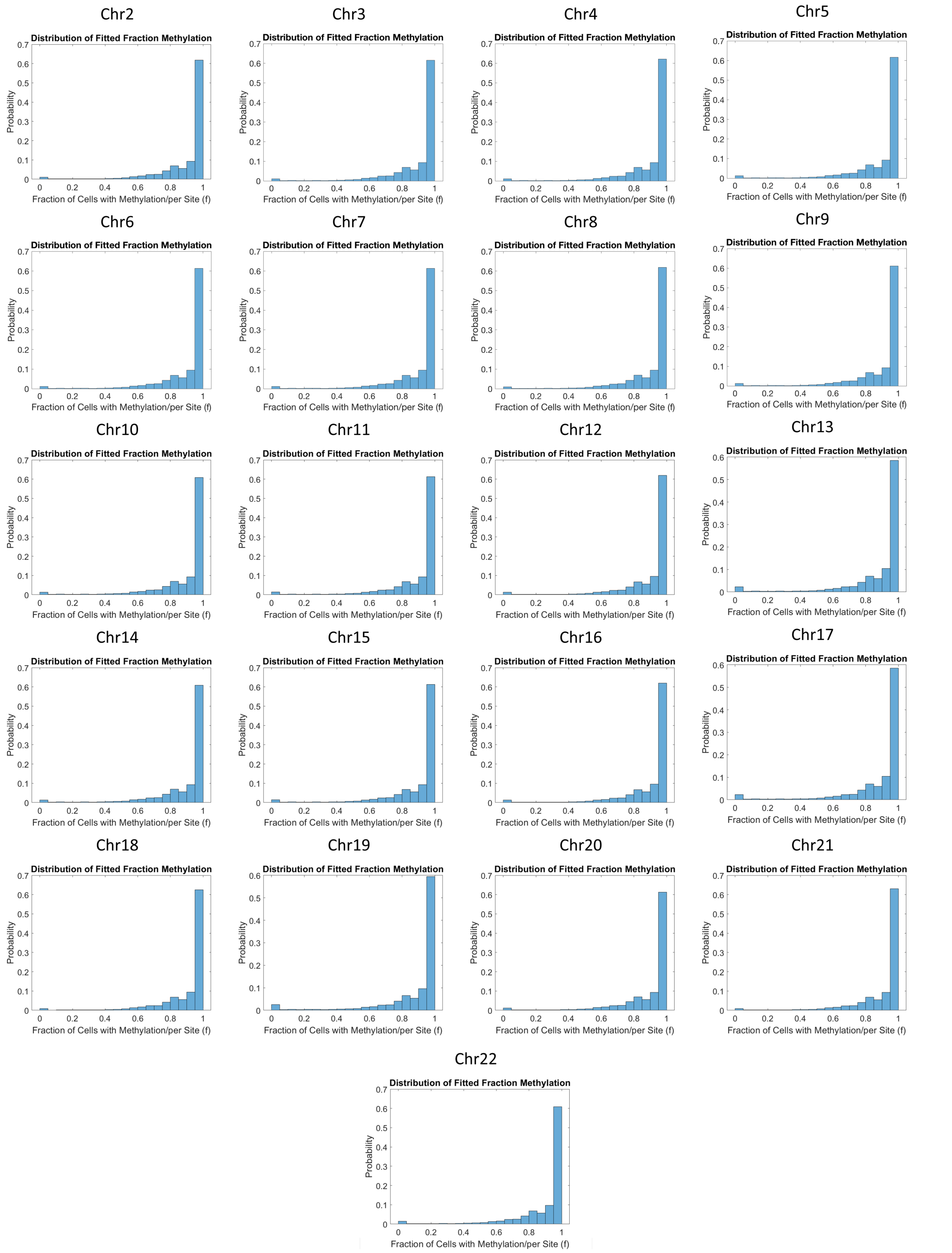

Fig I. Steady-state fraction methylation for Chromosomes 2 to 22. Histograms are normalized by probability.

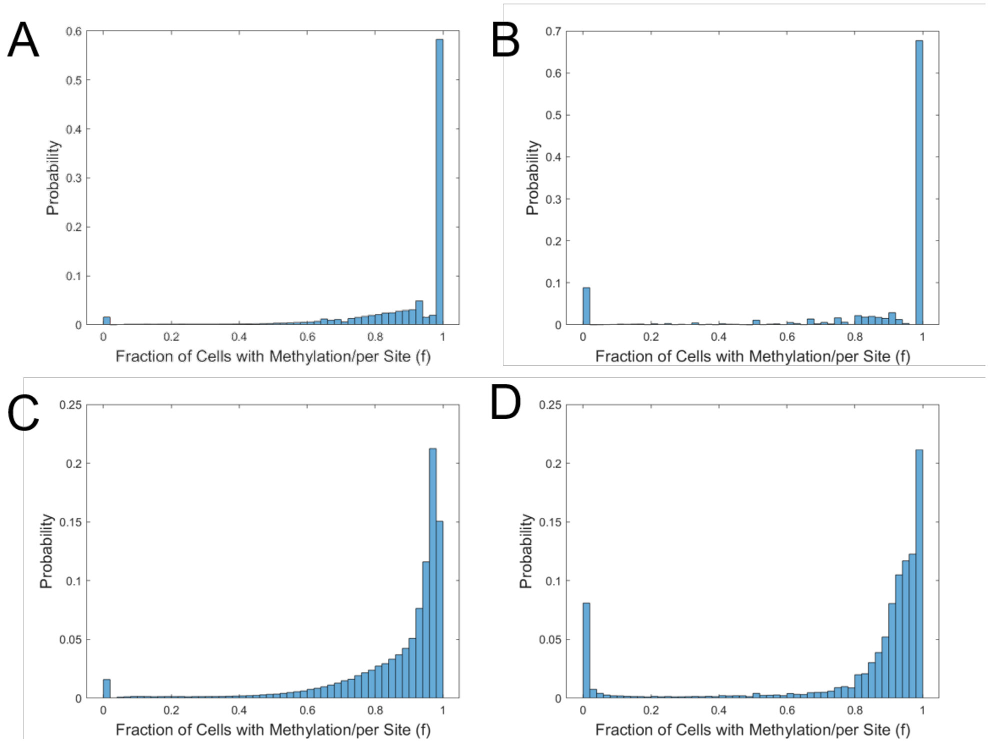

Fig J. Methylation fractions for Chr1. **A**:Steady-state fraction methylation as inferred by MLE. **B**: Methylation fraction from an independent experimental dataset of WGBS measurements from Chr1 in arrested HUES64 cells [1].**C**: Time-averaged methylation fraction for each site integrating Eq. 2 (Main Text) over 16.5 hr. **D**: Methylation fraction from an independent experimental dataset of WGBS measurements from Chr1 in proliferating HUES64 cells [1]. Histograms are normalized by probability.

Correlation of k with CpG_d for other chromosomes

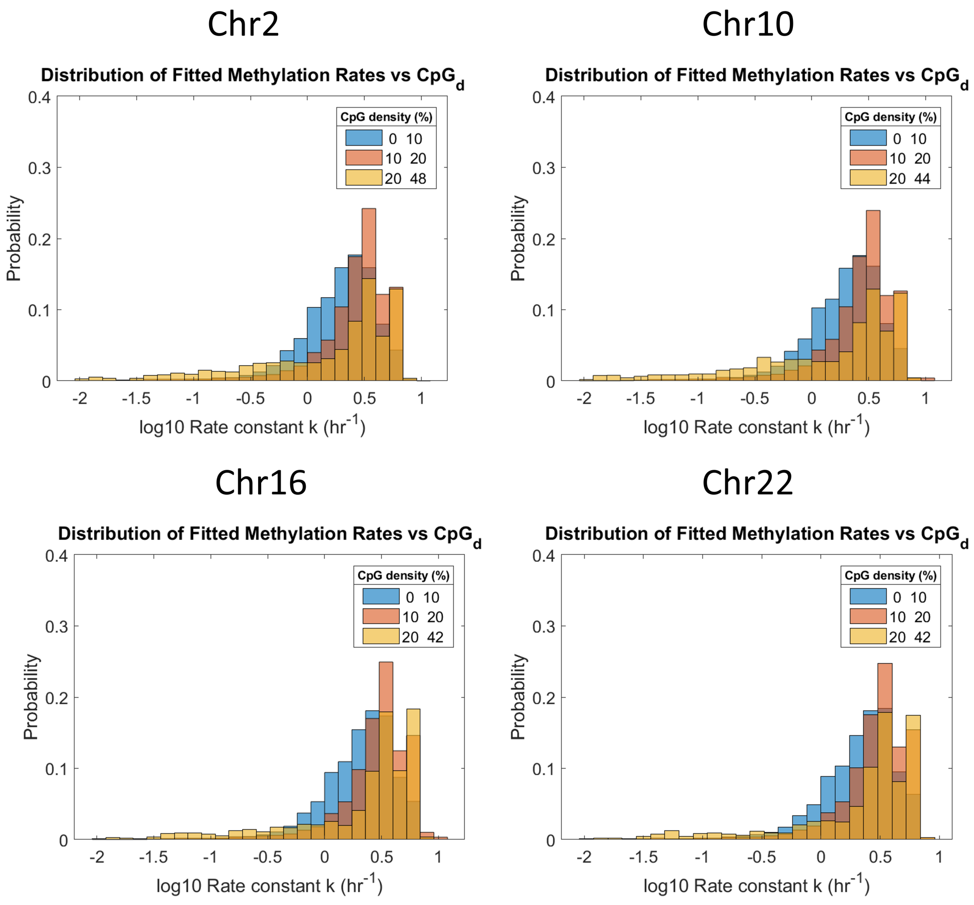

Fig K. Remethylation rates distributions for low (blue), medium (orange), and high-density (yellow) CpG sites of chromosomes 2, 10, 16, and 22. CpG-density of a site i is determined as the fraction of bp that are part of a CpG dinucleotide within a radius of 50 bp upstream and downstream the DNA molecule. Low density is defined as [0,10)%. Medium density is defined as [10,20)%. High density is defined as [20, $CpG_{d_{max}}$]%, where $CpG_{d_{max}}$ is the maximum CpG_d found in each chromosome (48, 44, 42, and 42% for Chr 2, 10, 16, and 22, respectively).

Remethylation rates distributions for low, medium, and high-density CpG sites with fraction methylation $f < 0.9$ and $f > 0.9$ for Chr1

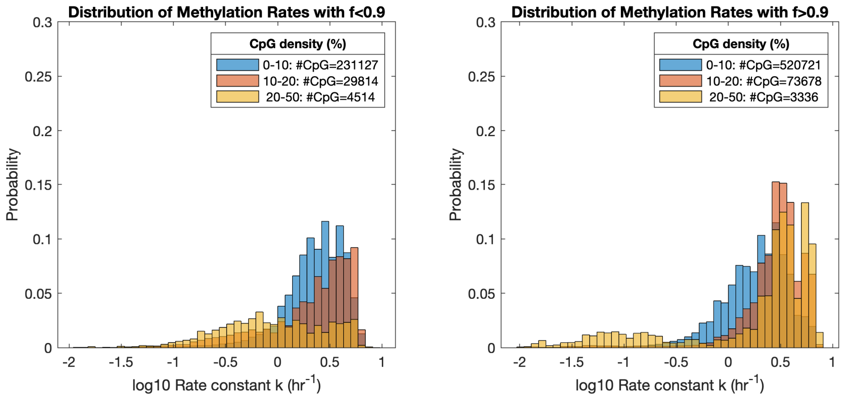

Fig L. Remethylation rates distributions for low, medium, and high-density CpG sites of Chr1 with fraction methylaition $f < 0.9$ (left) and $f > 0.9$ (right). Remethylation rates distributions with the same meaning as the last figure. CpG density of a site i is determined as the fraction of bp that are part of a CpG dinucleotide within a radius of 50 bp upstream and downstream the DNA molecule.

Correlation of f with CpG_d for other chromosomes

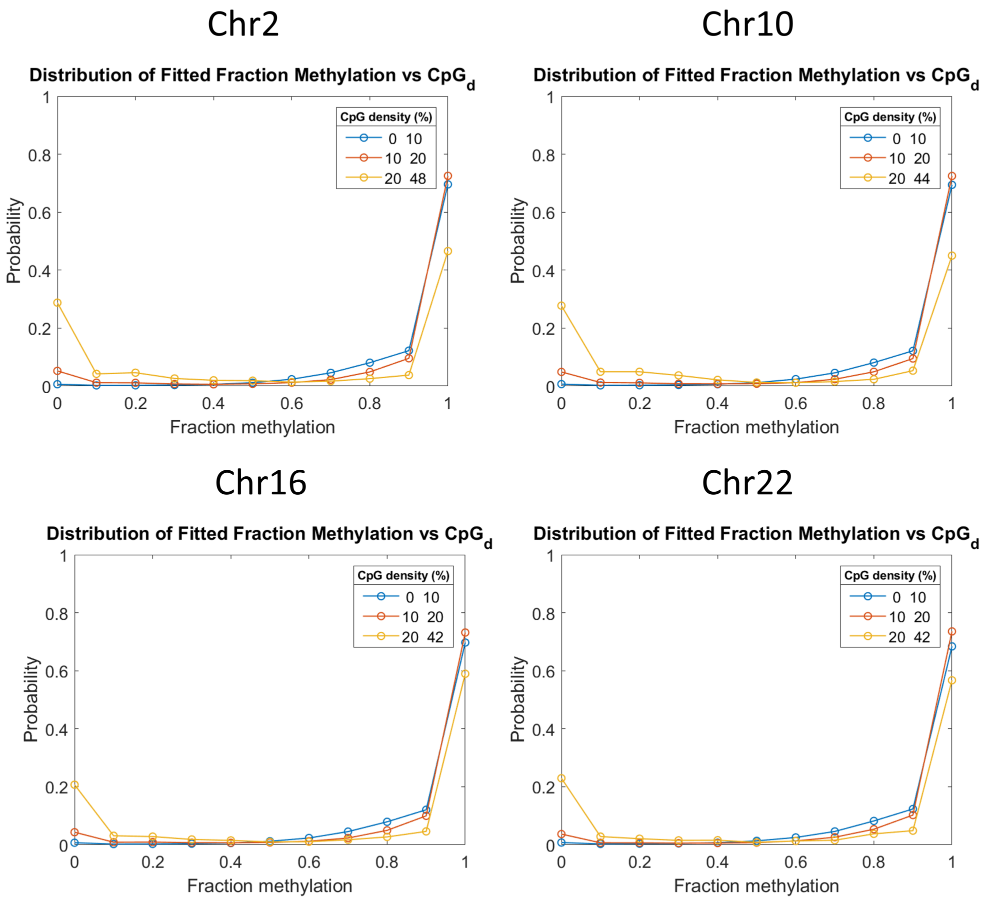

Fig M. Fraction methylation distributions for low (blue), medium (orange), and high-density (yellow) CpG sites of chromosomes 2, 10, 16, and 22. CpG-density of a site i is determined as the fraction of bp that are part of a CpG dinucleotide within a radius of 50 bp upstream and downstream the DNA molecule. Low density is defined as [0,10)%. Medium density is defined as [10,20)%. High density is defined as [20, $CpG_{d_{max}}$]%, where $CpG_{d_{max}}$ is the maximum CpG_d found in each chromosome (48, 44, 42, and 42% for Chr 2, 10, 16, and 22, respectively).

Correlation of k with GD for other chromosomes

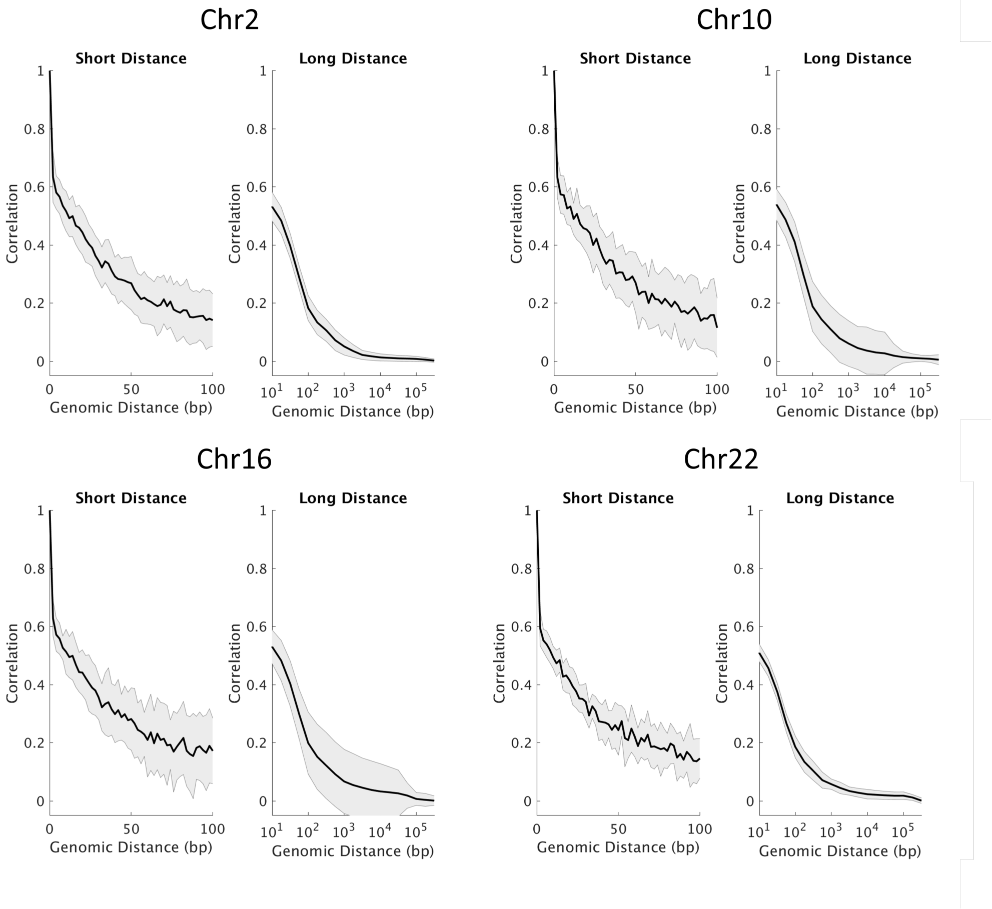

Fig N. Correlation of remethylation rates k with genomic distance for chromosomes 2, 10, 16, and 22. Correlation over short distances (left) and long distances (right)

Correlation of f with GD for other chromosomes

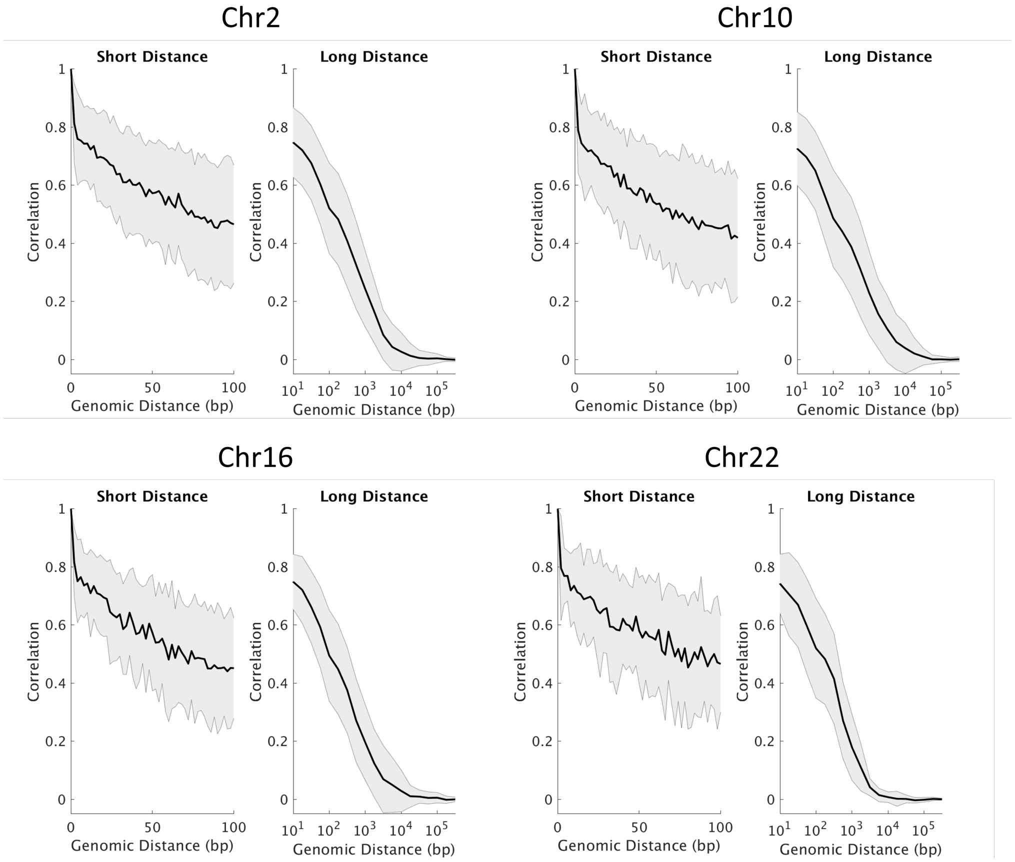

Fig O. Correlation of fraction methylation f with genomic distance for chromosomes 2, 10, 16, and 22. Correlation over short distances (left) and long distances (right)

Correlation function of categorized remethylation rates k for Chr1

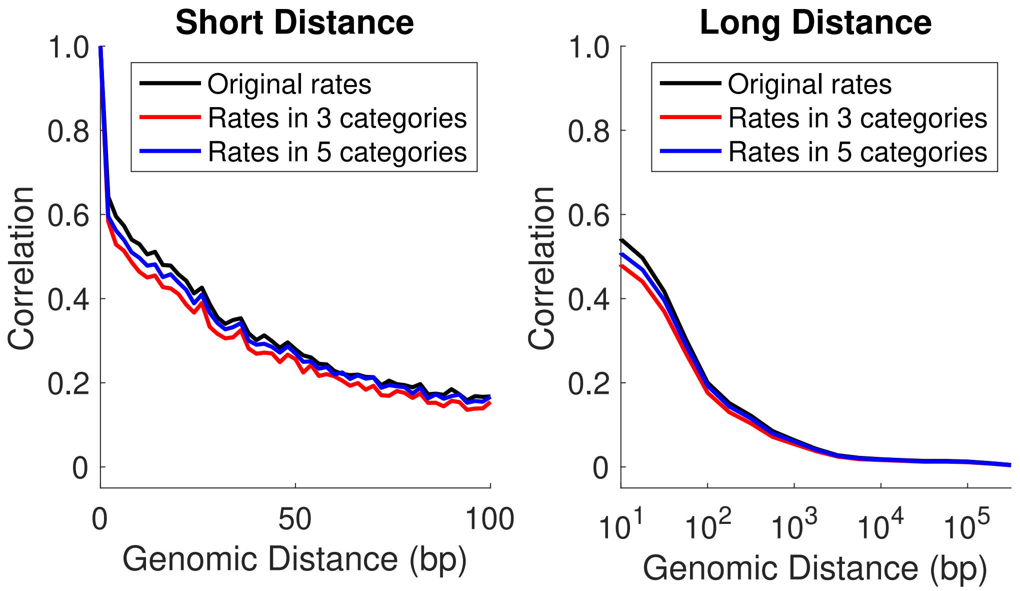

Fig P. Correlation function of remethylation rates k over short distances (left) and long distances (right) for Chr1. The black line is the correlation function of original methylation rates, whereas red and blue lines are the correlation functions for categorical rates, i.e. rates are discretized into 3 (red) and 5 categories (blue) by equally partitioned percentiles.

Histogram of 200bp tiled remethylation rates k and methylation fraction f of Chr1

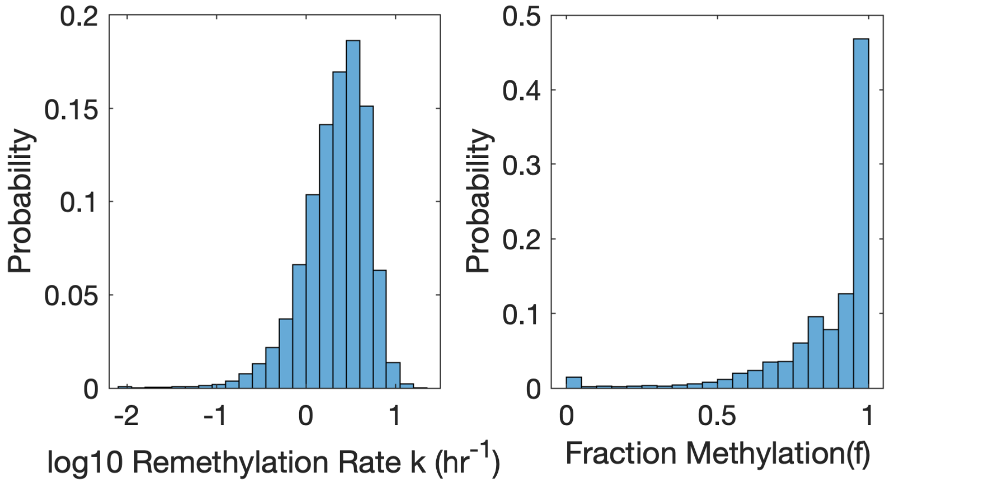

Fig Q. Histogram of 200bp tiled remethylation rates k (left) and methylation fraction f(right) of Chr1 The reads data from Repli-BS data are first summed within each non-overlapped 200bp tile across genome. Then, the remethylation rates k and methylation fraction f are inferred using this tiled data.

Correlation function of 200bp tiled remethylation rates k and methylation fraction f of Chr1

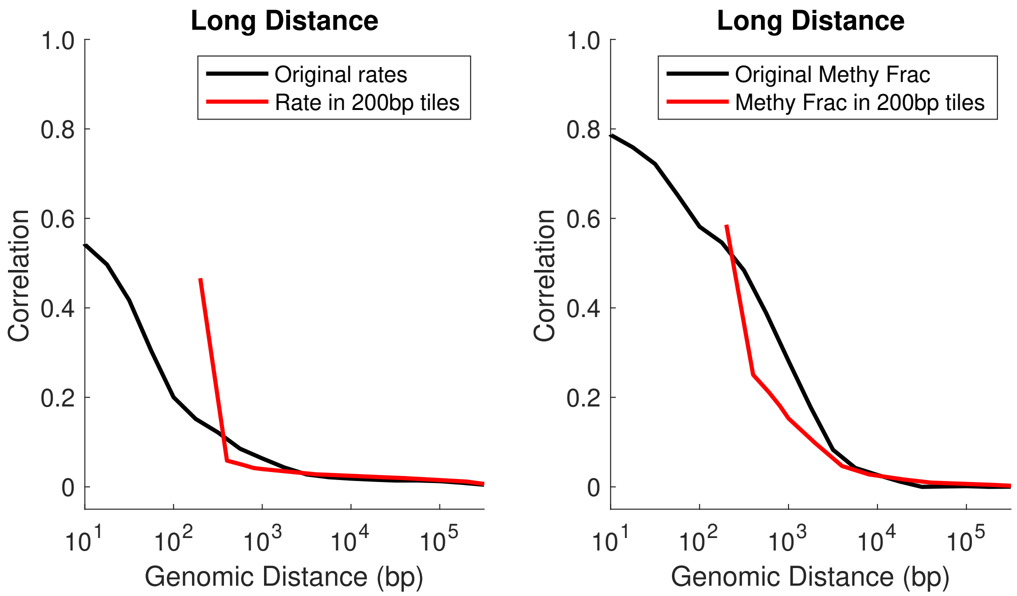

Fig R. Correlation function of 200bp tiled log(10) remethylation rates k (left) and methylation fraction f(right) Chr1.

Histogram of remethylation rates k and fraction methylation f for CpGs with different S-phase timing of Chr1

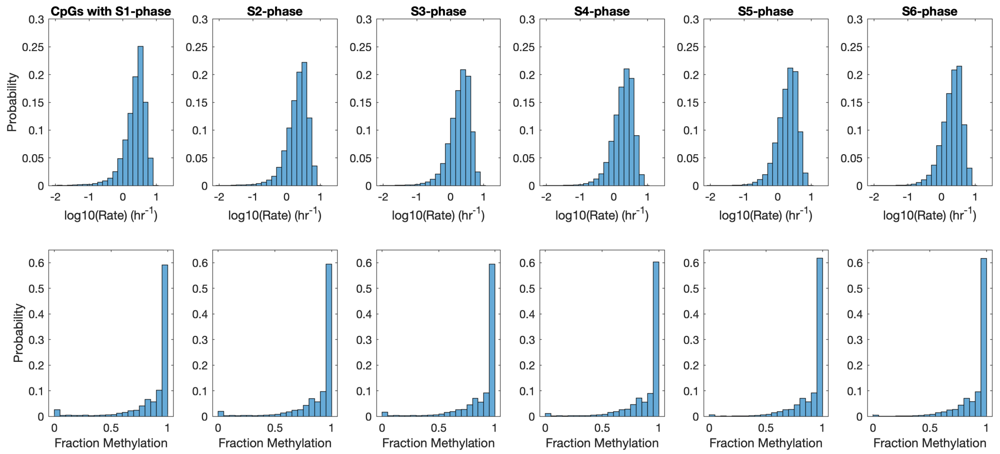

Fig S. Histogram of remethyaltion rates k (1st row) and fraction methylation f (2nd row) for CpGs with different S-phase timing

Correlation of remethylation rates k for CpGs with different S-phase timing of Chr1

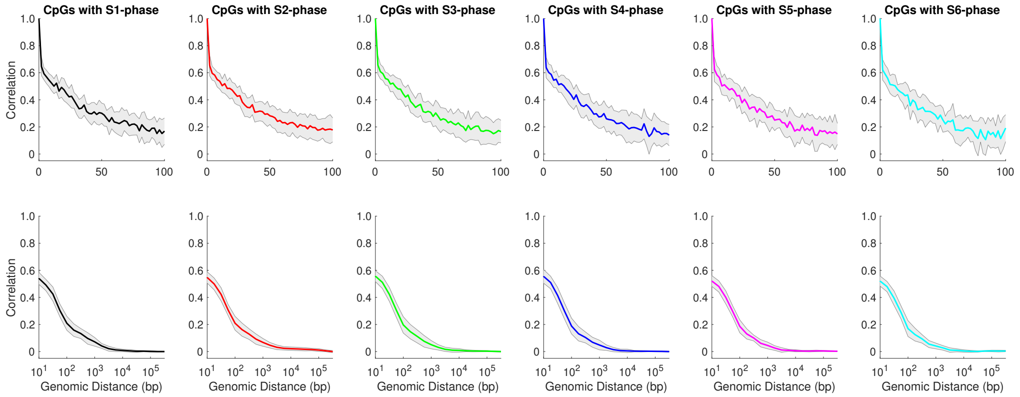

Fig T. Correlation of remethylation rates k for CpGs with different S-phase timing over short distances (1st row) and long distances (2nd row) of Chr1. The correlation function of newly synthesized DNA regions in different proportions of S-Phases are calculated. (See the definition of S-Phase proportions at Figure 1 in [1])

Correlation of fraction methylation f for CpGs with different S-phase timing

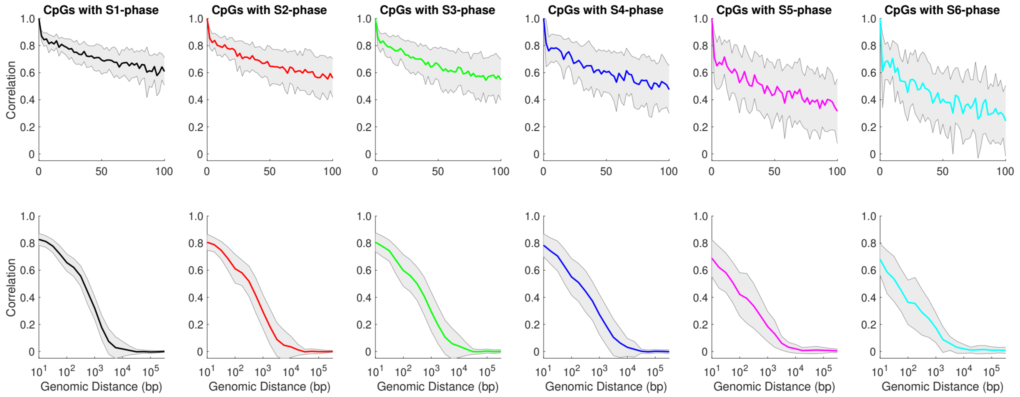

Fig U. Correlation of fraction methylation f for CpGs with different S-phase timing of Chr1

Correlation of remethylation rates k for CpGs in different genomic context of Chr1

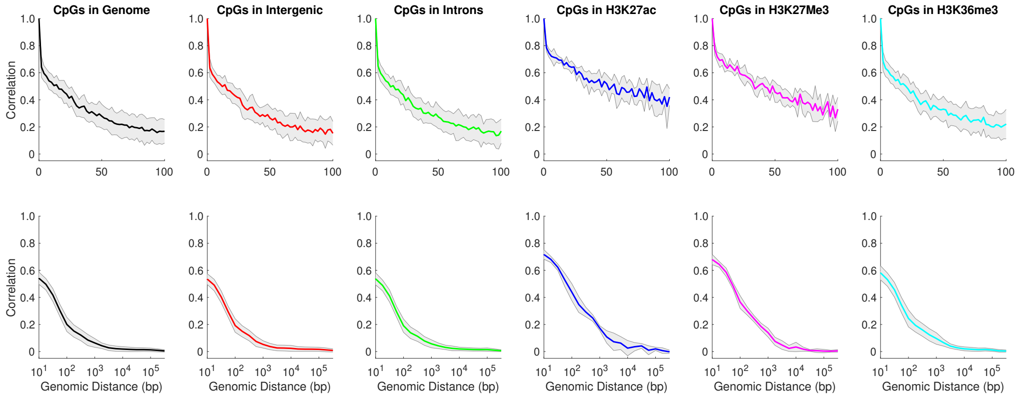

Fig V. Correlation of remethylation rates k for CpGs in different genomic context of Chr1 Here the genomic annotations are extracted from hg19 UCSC gene annotations. The histone modification peaks are downloaded from ENCODE/Broad Institute in hg19.

Correlation of fraction methylation f for CpGs in different genomic context of Chr1

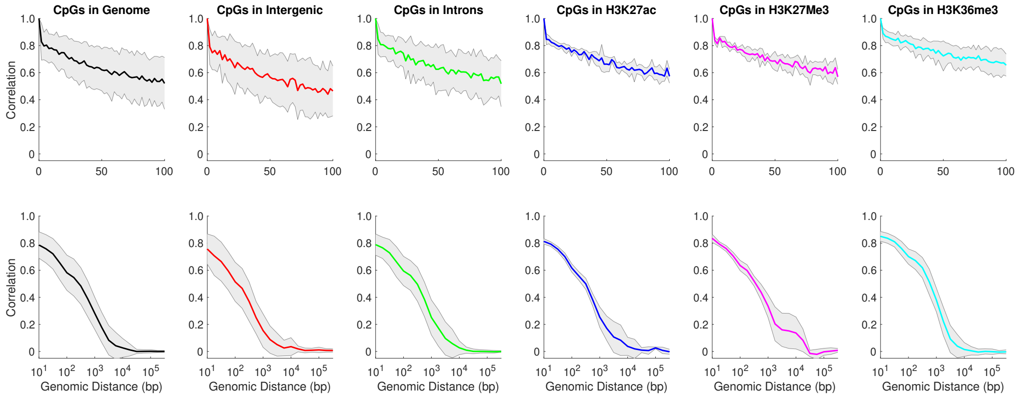

Fig W. Correlation of fraction methylation f for CpGs in different genomic context of Chr1

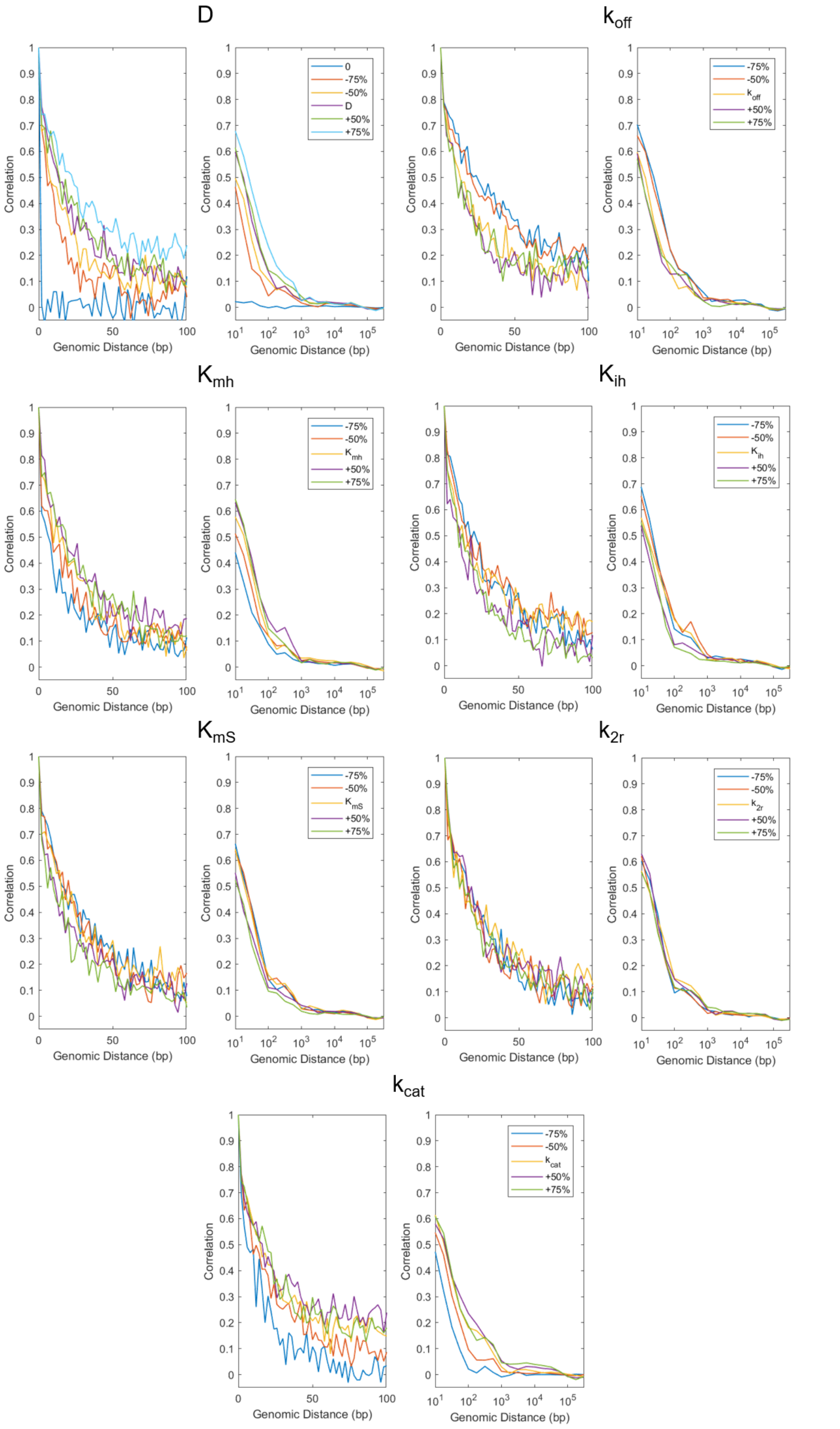

Fig X. Changes on the correlation function of k_{model} with genomic distance when varying different free parameters of the Processive model. 10^4 sites where used for each simulation. While some parameters clearly affect the correlation distance and the shape of the correlation function, others do not exert any influence within the tested range. Central values for each parameter are displayed in Table A.

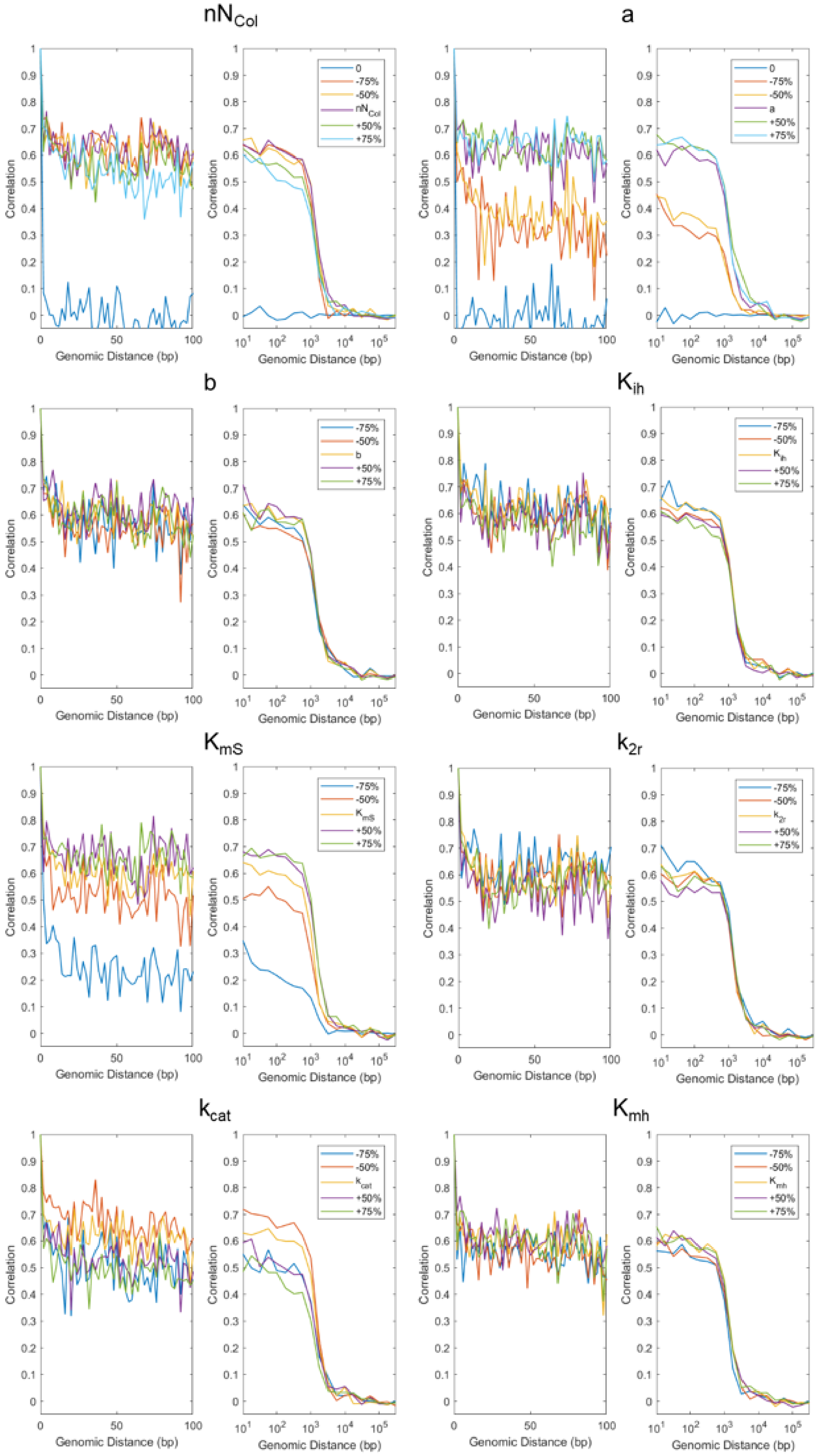

Fig Y. Changes on the correlation function of k_{model} with genomic distance when varying different free parameters of the Collaborative model. 10^4 sites where used for each simulation. While some parameters clearly affect the correlation distance and the shape of the correlation function, others do not exert any influence within the tested range. Central values are displayed in Table A.

While a deep understanding of the effect of each parameter on the obtained correlation functions is beyond the scope of this work, some of the observed changes can be explained in the light of the mechanistic aspects of the Processive model. Namely, when setting D (the 1D-diffusion coefficient of DNMT1 along DNA) to 0, the correlation function drastically tends to 0 (Fig. X), because the propensity of DNMT1 to diffuse towards a neighboring site, defined as k_{Dif}, is set to 0. This is also observed for $nDist$ (i.e the maximum distance DNMT1 can travel in a processive way). When set to 0 bp, no processive reactions can take place, and correlation indeed disappears. These observations reveal that k_{model} correlation with GD observed for the Processive model can be attributed to the set of processive reactions, rather than other reactions the model shares with the Distributive mechanism.

In the case of the Collaborative mechanism (Fig. Y), it can be observed how the correlation function of k_{model} and GD is in general insensitive to changes on many of the model's parameters, such as nN_{col} or k_{2r}, while being sensitive to parameters such as K_{mS} or a. Interestingly enough, correlation is absent when a is set to 0, for the propensity of any recruiting reaction (defined as k_{rec}) is null (See Eq. 13 in the main text). This is also observed for nN_{col} (i.e the furthest neighbor onto which recruitment can occur). When set to 0, no collaborative reactions are considered, and correlation again disappears. Thus, in a similar way to the Processive mechanism with the processive reactions, k_{model} correlation with GD for the Collaborative model can be attributed to the set of recruitment reactions the mechanism incorporates.

References

1. Charlton J, Downing T, Smith Z, Gu H, Clement K, Pop R, et al. Global delay in nascent strand DNA methylation. Nature Structural & Molecular Biology. 2018;25(4):327–332.

2. Cornish-Bowden A. Fundamentals of Enzyme Kinetics. 4th ed. Wiley-Blackwell, editor; 2012.

3. Wu JC, Santi DV. Kinetic and catalytic mechanism of HhaI methyltransferase. Journal of Biological Chemistry. 1987;doi:10.1016/S0959-440X(97)80013-9.

4. Pradhan S, Bacolla A, Wells RD, Roberts RJ. Recombinant Human DNA (Cytosine-5) Methyltransferase. Journal of Biological Chemistry. 1999;274(46):33002–33010. doi:10.1074/jbc.274.46.33002.

5. Milo R. B10Numbe3R5; 2010. Available from: `https://bionumbers.hms.harvard.edu/search.aspx`.

6. Smith ZD, Meissner A. DNA methylation: Roles in mammalian development; 2013.

7. Svedruzic Z[U+FFFD] Reich NO. Mechanism of allosteric regulation of Dnmt1's processivity. Biochemistry. 2005;44(45):14977–14988. doi:10.1021/bi050988f.

8. Tafvizi A, Huang F, Leith JS, Fersht AR, Mirny LA, Van Oijen AM. Tumor suppressor p53 slides on DNA with low friction and high stability. Biophysical Journal. 2008;95(1):1–3. doi:10.1529/biophysj.108.134122.

9. Wang YM, Austin RH, Cox EC. Single Molecule Measurements of Repressor Protein 1D Diffusion on DNA. Physical Review Letters. 2006;97(4):048302. doi:10.1103/PhysRevLett.97.048302.

10. Hermann A, Goyal R, Jeltsch A. The Dnmt1 DNA-(cytosine-C5)-methyltransferase Methylates DNA Processively with High Preference for Hemimethylated Target Sites. Journal of Biological Chemistry. 2004;279(46):48350–48359. doi:10.1074/jbc.M403427200.

11. Gillespie DT. Exact stochastic simulation of coupled chemical reactions. The Journal of Physical Chemistry. 1977;81(25):2340–2361. doi:10.1021/j100540a008.
